# Curcumin promotes cholesterol efflux from brain through LXR/RXR-ABCA1-apoA1 pathway in chronic cerebral hypoperfusion aging-rats

**DOI:** 10.1186/1750-1326-7-S1-S7

**Published:** 2012-02-07

**Authors:** Mingyuan Tian, Linhui Wang, Gang Yu, Bin Liu, Yu Li

**Affiliations:** 1 Department of Pathology, Chongqing Medical University, Chongqing 400016, China; 2 Institute of Neuroscience, Chongqing Medical University, Chongqing 400016, China; 3 Chongqing Key Laboratory of Neurobiology, Chongqing Medical University, Chongqing 400016, China; 4 Department of Neurology, The First Affiliated Hospital, Chongqing Medical University, Chongqing 400016, China

## Background

Chronic cerebral hypoperfusion has been well depicted as a common pathological status contributing to neurodegenerative disease such as vascular dementia (VaD) and Alzheimer’s dementia (AD). Cholesterol is critical to brain growth, but high levels of cholesterol have been associated with neurogenerative disease. The liver X receptor-β/retinoid X receptor –α (LXR/RXR)-regulated gene ABCA1 effluxes cellular cholesterol to apolipoprotein A1 (apoA1), which play important role in reverse cholesterol transport. Recent studies have found that Curcumin decrease brain inflammation and exert neuroprotective effect. We hypothesized that Curcumin may alleviate hypoperfusion through affecting cholesterol homeostasis by LXR/RXR-ABCA1-apoA1 pathway.

## Method

Male Sprague-Dawley rats were subjected to permanent occlusion of bilateral common carotid arteries (2VO) to produce chronic cerebral hypoperfusion. Animals were randomly divided into 5 groups: normal control group, sham-operated group, 2VO group, 2VO+Curcumin100mg/kg group, 2VO+ Curcumin50mg/kg group. Low doses of Curcumin (50mg/kg) or high doses of Curcumin (100mg/kg) were dissolved in DMSO. All animals were injected intraperitoneally with DMSO solution of Curcumin or a same volume of normal DMSO after surgery. Each group was injected once daily for four consecutive weeks. The spatial learning capacity and cognitive function of these animals was assessed in the Morris water maze 30 days after the onset of 2VO. The expressions of LXR/RXR, ABCA1 and apoA1 in hippocampus were detected by western blot and immunohistochemistry.

## Result

Spatial learning in the Morris water maze was significantly improved by the treatment of Curcumin comparing to 2VO group (p<0.05). Meanwhile, LXR-β/RXR-α, ABCA1 and apoA1 protein levels were increased in 2VO+Curcumin group (p<0.05). Interestingly, 2VO+Curcumin group had higher serum HDL cholesterol levels and total cholesterol (TC) levels than that of 2VO group (p<0.05). However, there were no statistical differences among 2VO+Curcumin group rats, sham-operated group rats and normal control group rats (p>0.05).

## Conclusion

We suggest that Curcumin have a protective influence on spatial learning in 2VO group rats. We also suppose that Curcumin may act a role of LXR agonist and then activate the ABCA1 promoter and increase ABCA1 protein levels and apoA1 dependent cellular cholesterol efflux from brain. This is the first study to show that Curcumin can alleviate hypoperfusion by LXR/RXR-ABCA1-apoA1 pathway.

